# Technology for the treatment promotion of adults living with HIV: *Positive o Cuidado* (Positive the Care)

**DOI:** 10.1590/0034-7167-2022-0454

**Published:** 2023-11-10

**Authors:** Vivian Costa Fermo, Francis Solange Vieira Tourinho, Douglas Dyllon Jerônimo de Macedo, Thaís Favero Alves

**Affiliations:** I Universidade Federal de Santa Catarina. Florianópolis, Santa Catarina, Brazil; II University of Nebraska Medical Center. Omaha, Nebraska, Estados Unidos da América

**Keywords:** Medication Adherence, Adult, Technology, Software Design, Human Immunodeficiency Virus, Cumplimiento de la Medicación, Adulto, Tecnología, Diseño de *Software*, Virus 1 de la Inmunodeficiencia Humana, Adesão à Medicação, Adulto, Tecnologia, Design de Software, Vírus da Imunodeficiência Humana

## Abstract

**Objectives:**

to develop a responsive website focused on treatment adherence for adult users living with HIV.

**Methods:**

technological study conducted between August and October 2020, in the light of Pierre Lévy’s theoretical-philosophical framework, using the Double Diamond Process methodology associated with the five stages of The Elements of User Experience framework.

**Results:**

it was developed the responsive website Positive Care (*Positive o Cuidado*), composed of an initial presentation screen and 13 other screens named: Family Health and You; Undetectable = Untransmissible; Antiretroviral Drugs; Routine Tests; Vaccination; Antiretroviral Delivery; Drug Interactions; Combined Prevention; Support Services; Healthy Life; Family and Reproductive Planning; Covid 19; and Questions, Curiosities, and Myths.

**Final Considerations:**

the responsive website was developed based on the software design and programming process and has requirements/functionalities with the potential to strengthen the collective intelligence about HIV and, consequently, to promote treatment adherence by its users.

## RESUMO

**Objetivos:**

desenvolver *site* responsivo com foco na adesão ao tratamento de usuários adultos vivendo com HIV.

**Métodos:**

estudo tecnológico realizado entre agosto e outubro de 2020, à luz do referencial teórico-filosófico de Pierre Lévy, usando a metodologia *Double Diamond Process* associada aos cinco planos do *framework The Elements of User Experience*.

**Resultados:**

foi desenvolvido o *site* responsivo “Positive o Cuidado”, composto por tela inicial de apresentação e mais 13 telas denominadas: Saúde da família e você; Indetectável = Intransmissível; Medicações antirretrovirais; Exames de rotina; Vacinação; Entrega de antirretrovirais; Interações medicamentosas; Prevenção combinada; Serviços de apoio; Vida saudável; Planejamento familiar e reprodutivo; Covid-19; e Dúvidas, Curiosidade e Mitos.

**Considerações Finais:**

o *site* responsivo foi desenvolvido com base no processo de *design* e programação do *software* e possui requisitos/funcionalidades com potencial de fortalecer a inteligência coletiva acerca do HIV e, consequentemente, promover a adesão ao tratamento por seus usuários.

## RESUMEN

**Objetivo:**

desarrollar sitio responsivo con enfoque en la adhesión al tratamiento de usuarios adultos viviendo con VIH.

**Métodos:**

estudio tecnológico realizado entre agosto y octubre de 2020, basado en el referencial teórico-filosófico de Pierre Lévy, usando la metodología *Double Diamond Process* relacionada a los cinco planes del *framework The Elements of User Experience*.

**Resultados:**

fue desarrollado el sitio responsivo “Positiva el Cuidado”, compuesto por pantalla inicial de presentación y miás 13 pantallas denominadas: Salud de la familia y tú; Indétectable = Intransmisible; Medicaciones antirretrovirales; Exámenes de rutina; Vacunación; Entrega de antirretrovirales; Interacciones medicamentosas; Prevención combinada; Servicios de apoyo; Vida saludable; Planeamiento familiar y reproductivo; Covid-19; y Dudas, Curiosidad y Mitos.

**Consideraciones Finales:**

el sitio responsivo fue desarrollado basado en el proceso de diseño y programación del *software* y posee requisitos/funcionalidades con potencial de fortalecer la inteligencia colectiva acerca del VIH y, consecuentemente, promover la adhesión al tratamiento por sus usuarios.

## INTRODUCTION

After learning about their serologic status, people living with human immunodeficiency virus (HIV) seek support on the Internet because they identify this space as comfortable, free of stigma and prejudice; and where they can exchange confidences, fears, and experiences that encourage them to face adversities. In addition, they search for information related to health, such as the use and effects of antiretroviral therapy (ART)^(1)^, routine tests, and healthy lifestyle habits, as well as support and instrumentalization in decision-making.

Given this reality, web-based health technologies (e-Health) have been implemented with a positive impact on ART adherence and the reduction of health risks. A systematic review aimed to assess the feasibility and impact of digital innovations in HIV and sexually transmitted infections (STI) care identified and evaluated 99 studies, of which used 69 innovations like mHealth (Short Message Service [SMS] and/or phone calls), 21 used e-health, and nine combined the two modalities. Only one study in Brazil addressing the use of SMS by women living with HIV^(2)^ demonstrates the gap in knowledge on this topic in the Brazilian scenario.

When planning the development of eHealth technologies, one must consider that, currently, the use of virtual environments through mobile devices is widespread. Among the operating systems, there is Android, used by several cellphone manufacturers, and those present a significant variation in screen format and definition; IOS, from Apple, is installed only in devices produced by the brand, favoring standard screens and interfaces^(3)^.

Due to this diversity of formats, the cyberspace content developer must consider how to offer the best user experience. The responsive site solves that challenge since it is a group of web pages with a layout compatible with all screen sizes of cell phones, tablets, and computers, which leads to a good resolution and usability, as well as the organization of its elements according to the device^(3)^. In addition, healthcare professionals can create, implement, and maintain responsive websites, allowing them to provide care to people living with HIV in an easy-to-maintain, low-cost technology design.

## OBJECTIVES

To develop a responsive website focused on treatment adherence for adult users living with HIV.

## METHODS

That is a technological study contemplating the development of technological innovation (responsive website). It is part of one of the stages of the doctoral thesis entitled Positive Care: responsive website for treatment adherence of adult users living with HIV (*Positive o Cuidado: site responsivo para a adesão ao tratamento de usuários adultos vivendo com HIV*) of the Nursing Graduate Program of the Federal University of Santa Catarina. The research followed the precepts of the CNS resolution n. 466/2012 with the approval of the Ethics Committee on Research with Human Beings.

It is well known that software engineering has four fundamental development activities: specification; development; validation; and evolution^(4)^. The present study reports the activity “software development,” carried out between August and October 2020, composed of the phases: the design process and software programming. Thus, the design process of the responsive website “Positive Care” was carried out using the Double Diamond Process methodology^(5)^ associated with the five plans defined by the framework called The Elements of User Experience, developed by Garret^(6)^, containing the plans: strategy, scope, structure, skeleton, and surface^(6)^.

**Figure 1:**
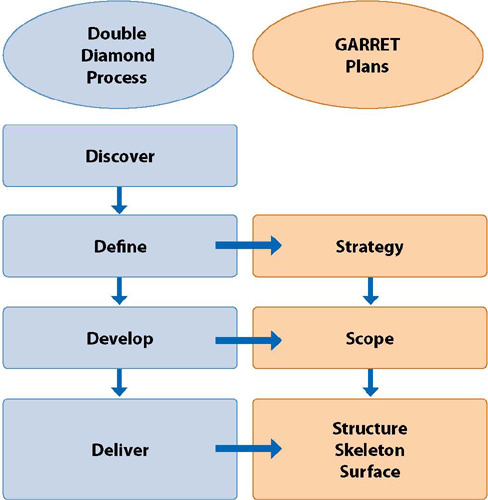
Relations of the Double Diamond Process with the elements of User Experience in the design process of the Positive Care responsive website, Florianópolis, Santa Catarina, Brazil, 2022

The Double Diamond Process explores a problem through divergent thinking processes and, in sequence, convergent action, allowing, at the end of each stage (discover, define, develop, scope and deliver), to solve problems and, if necessary, return to the previous stage^(5)^. Still, throughout the process, the researcher and the designer exchanged ideas to think and test the solutions, refining the products until they reached the desired goal. The association of the Double Diamond Process to the elements of User Experience is represented in Figure 1.

In the “Discover” stage, there was a briefing between the design professional and the researcher to discuss the objective, the target audience of the project, and the need for research identified regarding HIV portals, health area portals, content aggregator sites, and portals, use of color and typography in the health area. In the “Define” stage, we selected the ideas most aligned or best represented the portal’s objectives. In this phase, the “Strategy” plan took place, with the strategic definition of the user experience elements and definitions of the desires of those who carry out the project (researcher), which included meeting the needs of the technology users (user requirements) and other stakeholders. It is noteworthy that the needs of users and other stakeholders were identified in the software specification stage, in which the researcher held brainstorming sessions with people living with HIV and health professionals (nurses, doctors, and pharmacists) who provide health care to these people in Primary Health Care and the Specialized Care Service (SAE) of a municipality in the state of Santa Catarina.

The “Develop” stage was the time to test possibilities, draw initial sketches, build possibilities, and create based on the scope definitions. The typographic family and colors were implemented as a navigation resource, division of areas, and content organization style. In this stage, the design professional receives the contents built based on current scientific evidence from the researcher. Next, the “Scope” plan took place, with the moment to close the scope of the portal and its specifics, such as being responsive, displaying video resources, text, images/illustrations, submission form, and contact tool.

The “Deliver” stage took place when the project began to take visual form and executed the plans “Structure,” “Skeleton,” and “Surface.” In the “Structure” plan, the flowchart and the architecture of the platform were elaborated using the Whimsical© tool; in the “Skeleton” plan, the wireframes, the context/study population, the data sources, the measurement instrument, the modality and period of data collection, the analysis processes, among other items, were defined. The interface and navigation used the Whimsical© tool. Finally, visual elements were defined in the “Surface” plan, and the visual identity was applied to the skeleton using Figma®.

After implementing the project’s design stages, with the interface’s construction and approval, we proceeded to the responsive website programming. The platform was developed with HTML, CSS, and PHP using the Wordpress® CMS. It is noteworthy that all software cited for developing this technology is free.

### Theoretical philosofical referencial

The theoretical-philosophical referential of Pierre Lévy developed the responsive website. It defines cyberspace as a means of communication through the interconnection of computers, housing the material infrastructure of digital communication, the information, and the people who interact in it^(7)^. Among the potentials of cyberspace are increasing the autonomy of individuals and fostering their cognitive faculties; improving collaboration among people; and stimulating collective intelligence, defined as intelligence that leads to the mobilization of competencies whose goal is the mutual recognition and improvement of people^(8)^.

Web-based information and communication technologies are a space for everyone and accept everyone; it is up to society, through the establishment of positive human relations^(7)^, to foster technodemocracy (the collective to participate in the deliberative debate about the technologies developed and applied) and collective intelligence; and to generate positive impacts from the use of technology^(9)^.

## RESULTS

**Figure 2:**
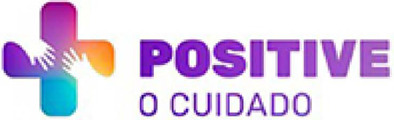
Logo of the responsive website “Positive the Care”, Florianópolis, Santa Catarina, Brazil, 2022

The responsive website, “Positive the Care.”, is available through the hyperlink www.positiveocuidado.com, composed of an initial presentation screen and 13 other screens that meet the functional requirements identified during the software specification stage. The name “Positive the Care” was chosen because, from the moment of a positive HIV test result and knowledge about the diagnosis, the person living with HIV needs to make choices that contribute to their care and result in a better quality of life, as well as reduce the risk of HIV transmission to sexual partners. We show the logo for the responsive website in Figure 2.

We used colors to represent diversity in the logo and responsive website and illustrations instead of images to avoid stereotypes. The main screen presents the technology, mission, and items (with an icon to access each item). Each one contains contents built based on the functional requirements. Videos, video animation, texts, images/illustrations, podcasts, and hyperlinks to other digital platforms are several tools approaching the contents: Table 1 shows the functional requirements met by this technology.

The responsive website enables the user to contact the person in charge of the platform, the nurse, and the researcher through a message application (WhatsApp®) and e-mail duvidas@positiveocuidado.com. Those means allow the user to send questions involving HIV to elaborate the podcasts indexed in the screen “Questions, Curiosities and Myths.”

By recognizing privacy as a fundamental right, the technology presents its Privacy and Cookies Policy, which aims to establish and share, in a transparent, objective, and straightforward way, how to gather users’ information and data, handle, store, and treat and protect.

The technology had its Computer Program Registration Certificate issued on 04/13/2021 by the Brazilian National Institute of Industrial Property under number BR512021000703-4.

## DISCUSSION

People living with HIV deal with the feeling of living with a chronic disease and, in this process, may show several reasons for abandoning treatment linked to living conditions, relationships, social support, social vulnerability, work, acceptance of the disease, side effects of ART and other factors of daily life. Treatment adherence is essential for quality of life and to block the virus’s transmission chain, being fundamental approaches directed to listening and guidance and family and social support, promoting the reception and strengthening of positive actions, overcoming and willingness to live with quality^(10)^. Thus, the complexity of factors surrounding treatment adherence appears in the functional requirements the developed technology meets by understanding that adherence to HIV treatment encompasses several aspects beyond the clinical aspects of the person living with the virus.

**Chart 1:** Items displayed on the screens, access link, and functional requirements met by that technology, Florianópolis, Santa Catarina, Brazil, 2022

**Screen 1: Family Health and You (http://positiveocuidado.com/saúde-da-familia-e-voce/)**
To make the Florianopolis e-mail and WhatsApp® contacts of the Family Health Strategy teams available to the user; to provide guidance on the treatment of HIV in the Basic Health Units and on the referral to a specialist, when necessary; to provide orientation on legislation, rights and social benefits of the person living with HIV; to address how to deal with prejudice during health care and in the workplace.
**Screen 2: Undetectable = Untransmissible (http://positiveocuidado.com/indetectavel-intransmissivel/)**
Provide motivational videos for adherence to treatment with stories from people living with HIV, with the possibility for the user of the technology to share their own experience; provide a place for people to tell their life stories, to give positive stories, in the face of the challenges imposed by HIV, without the need to identify themselves; provide guidance on “Undetectable = Untransmissible”.
**Screen 3: Antiretroviral medications (http://positiveocuidado.com/medicações-antirretrovirais/)**
Guiding on ART side effects, severity, and management.
**Screen 4: Routine Tests (http://positiveocuidado.com/exames-de-rotina/)**
Provide orientation about routine tests in HIV treatment, their periodicity and importance; provide the reference values of the tests.
**Screen 5: Vaccination (http://positiveocuidado.com/vacinação/)**
Orientation about vaccinations.
**Screen 6: Antiretroviral Delivery (http://positiveocuidado.com/entrega-de-antirretrovirais/)**
Inform the location of the antiretroviral drug dispensing units in Florianopolis, their opening hours, and contact details (telephone and e-mail).
**Screen 7: Drug Interactions (http://positiveocuidado.com/interações-medicamentosas/)**
Allow the user to select antiretroviral drugs and other drugs to obtain information about drug interactions; inform about drugs that cannot be used with antiretroviral drugs when performing drug interaction assessment.
**Screen 8: Combined Prevention (http://positiveocuidado.com/prevenção-combinada/)**
Providing guidance on combined prevention.
**Screen 9: Support Services (http://positiveocuidado.com/serviços-de-apoio/)**
Make available a list of locations in Florianópolis that provide free legal and psychological assistance.
**Screen 10: Healthy Life (http://positiveocuidado.com/vida-saudável/)**
Provide guidance on the adoption of healthy lifestyle habits, such as diet and physical activity; advise on the use of alcohol, tobacco, and other recreational drugs.
**Screen 11: Family and Reproductive Planning (http://positiveocuidado.com/planejamento-familiar-e-reprodutivo/)**
Address voluntary communication about the diagnosis to the partnership and guide the search for support from a health professional to evaluate strategies for communicating the diagnosis; guide serodiscordant couples about family planning and pregnancy.
**Screen 12: Covid-19 (http://positiveocuidado.com/covid-19/)**
Providing guidance on care during the covid-19 pandemic.
**Screen 13: Questions, Curiosities and Myths (http://positiveocuidado.com/duvidas-curiosidades-e-mitos/)**
Explain the difference between HIV and AIDS; provide question and answer platform on HIV related issues; address HIV related questions and myths.

*HIV – human immunodeficiency virus; ART – antiretroviral therapy.*

e-Health technologies (cellphone applications, websites, online campaigns, telenovela videos, avatar-driven computer programs) are known to be feasible and complementary for improving the prevention and care of HIV and other STIs; they have a positive impact on HIV treatment adherence and can be customized and contextualized for hard-to-access populations^(2)^.

Given the above and reflecting on the concepts of cyberspace, collective intelligence, and technodemocracy, brought by Pierre Levy^(7, 8, 9)^, which led this study, users of “Positive Care” can find information to help themselves develop skills for adherence to HIV treatment. They will interact with the technology, transforming themselves and being able to transform it by sending content (video or text), sending e-mails with suggestions for the podcasts, or contacting the person responsible for the site (nurse) to clarify questions as well as for suggestions, support or other demands they consider appropriate.

By viewing the users interacting in cyberspace as part of an environment where they can participate as active social actors, with their experiences, and make transformations, the responsive site can address the rights of people living with HIV and the mechanisms to ensure them. Moreover, it allows popular participation in developing the Unified Health System (SUS). It is also worth mentioning that the offer of welcoming spaces through e-Health to people living with HIV should be guaranteed by public health policies, which demands the insertion of the topic “internet and health” in the training part of health professionals^(10)^.

Positive Care” emerges, therefore, as a tool that offers that space the support to the treatment promotion of people living with HIV through different technological resources (audio, videos); and enables the reception of its users through access to the nurse responsible for the technology through message application and e-mail.

Moreover, technological innovations must be proven to have impact and cost-effectiveness through clinical, randomized, observational, or qualitative studies^(2)^. That said, while developed technology is available and used by its target audience, future studies with different methodological approaches should be implemented to analyze the impact of its results on the health of people living with HIV over time.

### Study limitations

Regarding the benefits of cyberspace, the constructed site needs to introduce more assistive tools, such as Brazilian Sign Language translation, in its videos, for example, to maximize the inclusion of all citizens as participating and active members in the building of collective intelligence.

### Contributions to the Fields of Nursing, Public and Health

The responsive website “Positive Care” has the potential to reinforce the collective intelligence about HIV treatment and, consequently, to promote its users’ treatment adherence. Health professionals can promote it among people living with HIV to whom they provide care, as well as to whom live with those people. Significantly, those living with people living with HIV can influence health-related decision-making and thus can become effective supporters of HIV treatment. Furthermore, a website that addresses the issues surrounding living with HIV and HIV treatment can support the learning of people living with HIV and their social supporters.

## FINAL CONSIDERATIONS

The development of a responsive website based on the process of design and software programming provides people living with HIV quick, easy, and anywhere (as long as connected to the internet) access to information based on current scientific evidence about issues involving living with HIV and its treatment. Through different devices (computers, tablets, and smartphones), it offers different browsing experiences but maintains the content and the pattern of its design. The availability of the messaging application facilitates the user’s communication with the site’s developer, ensuring their participation in the maintenance of the technology, meeting their needs, and following the current social reality.

The complexity of factors interfering with HIV treatment adherence is noticeable in the functional requirements met by the presented technology. Due to the complexity of HIV treatment, the responsive website alone cannot consolidate treatment adherence, but it becomes one more strategy to be added to the countless others that society can implement to contribute to and promote such adherence.

## References

[1] Souza FBA, Sampaio ACL, Gomes MP, Silva GA, Silva ALB, Almeida EB (2019). Changes in The Quotidian of Women Living With Hiv: Ambulatorial Analysis, Rio De Janeiro State, Brazil. Res. Fundam. Care.

[2] Daher J, Vijh Rohit, Linthwaite B, Dave S, Kim J, Dheda K (2017). Do digital innovations for HIV and sexually transmitted infections work? Results from a systematic review (1996-2017). BMJ Open.

[3] Steffen A (2020). tela dos dispositivos Android: um percurso de pesquisa sobre design responsivo. Projética.

[4] Sommerville I. (2011). Engenharia de Software.

[5] Design Council (2020). What is the framework for innovation? Design Council's evolved Double Diamond [Internet].

[6] Garrett JJ (2006). Customer Loyalty and the Elements of User Experience. Des Manage Rev.

[7] Lévy P. (2010). Cibercultura.

[8] Lévy P (2004). Inteligencia colectiva: por una antropología del ciberespacio.

[9] Lévy P (2010). As tecnologias da inteligência: o futuro do pensamento na era da informática.

[10] Neto AO, Camargo KR (2019). Internet and HIV/Aids: a virtual ethnography on Facebook. Interface (Botucatu).

