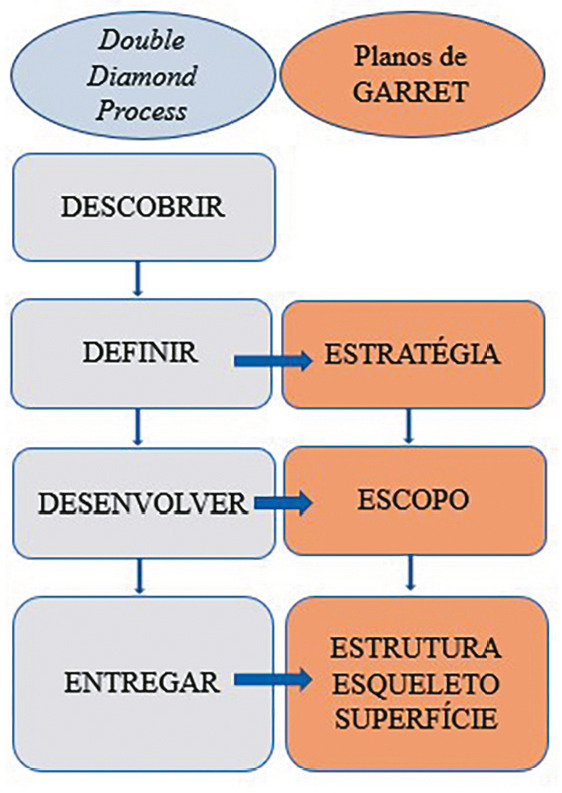# ERRATA

**DOI:** 10.1590/0034-7167.202578suppl4e07pt

**Published:** 2025-12-08

No artigo “Tecnologia para a promoção do tratamento de usuário adulto vivendo com HIV: Positive o cuidado”, com número DOI: https://doi.org/10.1590/0034-7167-2022-0454pt, publicado no periódico Revista Brasileira de Enfermagem, 2023;76(Suppl 4):e20220454, somente na versão em português, na página 2:

Onde se lia:

**Figure:**
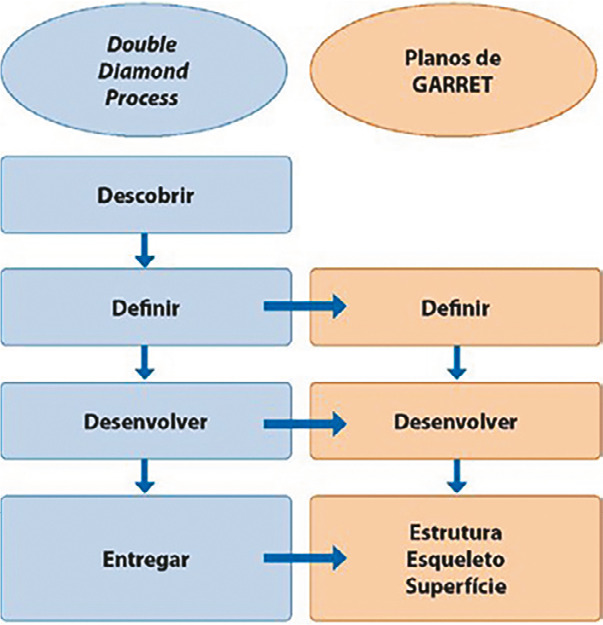

Leia-se:

**Figure:**